# Glutathione effect on functional and histological recovery after spinal cord injury in rats

**DOI:** 10.1016/j.clinsp.2024.100359

**Published:** 2024-04-23

**Authors:** Fernando Flores de Araujo, Raphael Martus Marcon, Alexandre Fogaça Cristante, Tarcísio Eloy Pessoa Barros Filho

**Affiliations:** a Instituto de Ortopedia e Traumatologia, Hospital das Clínicas, Faculdade de Medicina, Universidade de São Paulo (IOT-HCFMUSP), São Paulo, SP, Brazil; b Grupo de Cirurgia de Coluna, Laboratório de Investigações Médicas, Instituto de Ortopedia e Traumatologia, Hospital das Clínicas, Faculdade de Medicina, Universidade de São Paulo (IOT-HCFMUSP), São Paulo, SP, Brazil

**Keywords:** Glutathione, Spinal cord injuries, Central nervous system/injuries, Rats, Antioxidants/Drug effects

## Abstract

**Objective:**

The aim of this study was to evaluate the GSH effect on functional and histological recovery after experimental spinal cord injury in rats.

**Methods:**

Forty Wistar rats were subjected to spinal cord injury through the Multicenter Animal Spinal Cord Injury Study (MASCIS) Impactor system. The rats were sorted and divided into four groups, as follows: Group 1 ‒ Laminectomy and spinal cord injury; Group 2 ‒ Laminectomy, spinal cord injury and Saline Solution (SS) 0.9%; Group 3 ‒ Laminectomy, spinal cord injury, and GSH; and Group 4 ‒ lLaminectomy without spinal cord injury. GSH and SS were administered intraperitoneally. Groups 1 and 4 received no intervention.

**Results:**

The rats were evaluated for locomotor function recovery at seven different times by the Basso, Beattie, and Bresnahan (BBB) scale on days 2, 7, 14, 21, 28, 35, and 42 after the spinal cord injury. On day 42, the rats were sacrificed to analyze the histological findings of the injured spinal cord. In the group submitted to GSH, our experimental study revealed better functional scores on the BBB scale, horizontal ladder scale, and cranial and caudal axon count. The differences found were statistically significant in BBB scores and axonal count analysis.

**Conclusion:**

This study demonstrated that using glutathione in experimental spinal trauma can lead to better functional recovery and improved axonal regeneration rate in Wistar rats submitted to experimental spinal cord injury.

## Highlights

• Glutathione is important in protecting secondary spinal cord injury from oxidative stress.
• As the secondary injury progresses, depletion in its reduced form occurs.
• Using glutathione in traumatic spinal cord injury could help control the neurological injury.
• In an experimental setting, the use of glutathione provided better functional scores after traumatic spinal cord injury.

## Introduction

Spinal cord injury occurs by two mechanisms; primary injury is caused by direct trauma to the spinal cord, and secondary injury is a consequence of primary injury, resulting in a sequence of self-destructive biochemical events that can last for hours or days leading to dysfunction and cell death.^1^^,^^2^ These events are characterized by a pattern of progressive activation of several pathophysiological mechanisms, including microvascular perfusion changes, inflammation, the release of oxygen free radicals and lipid peroxidation of the cell membrane, deregulation of ion cell flow control, and cell death by apoptosis.^1^, ^2^, ^3^ In the acute phase, necrosis occurs in the central gray matter, mainly in the first hour after injury, followed by edema and hemorrhage in the following hours. This process results in ischemia caused by reduced blood flow to the affected spinal cord segment. This reduction in blood flow may be caused by a change in the vertebral canal, significant hemorrhage and edema of the spinal cord, or a decrease in systemic blood pressure. Ischemia creates a chain of biochemical reactions resulting in cell death. Then, inflammatory cells migrate to the injured site, and glial proliferation occurs simultaneously. The formation of scar tissue and cysts occurs in the chronic phase, in the period of one to four weeks, due to the proliferation of astrocytes and hypertrophy, forming a glial scar.^4^^,^^5^

Over the years, the attempt to obtain more effective treatment for spinal cord injury was based on four basic approaches: surgical, physical, biological, and pharmacological.^6^ In this context, research by pharmacological approach to control secondary injuries has gained prominence in the last decade.^7^ Pharmacological therapy has a promising role in preventing secondary injury. Neurons and glial cells from the central nervous system are particularly prone to oxidative and electrophilic stress due to many factors, including a high content of polyunsaturated fatty acids, a high rate of oxidative metabolic activity, intense production of reactive oxygen metabolites, and relatively low antioxidant capacity. Thus, increased oxidative stress is considered a marker of spinal cord injury.^3^

Glutathione (GSH) is a molecule produced by almost all cells of the body, with a fundamental role in maintaining the redox state of the cell and protecting it from oxidative stress. GSH has low molecular weight and still acts as a cosubstrate for several other antioxidant enzymes, such as GSH peroxidase and GSH transferase. The main cells of the nervous system, neurons, have relatively low levels of GSH compared to other organs, such as the liver, which makes them especially vulnerable to oxidative stress. On the other hand, GSH is present in high concentrations in astrocytes, glial cells that still provide substrates for synthesizing GSH in neurons. Neuros incubation with glial cells for 24 hours increased levels of GSH in the neurons. The main substrates for GSH synthesis are cysteine, glutamate, and glycine. In addition, GSH can also be an important neuromodulator of the central nervous system.^8^

The aim of this study is to evaluate, in a standardized model, the nerve regeneration and motor functional recovery of Wistar rats with surgically induced paraplegia in response to GSH.

## Material and methods

The study was approved by the Institution's ethics committee. The research protocol was defined according to the international guidelines for animal research and publication–Animal Research: Reporting of *In Vivo* Experiments (ARRIVE). Forty adult male Wistar rats, weighing from 267g to 412g, kept at a controlled temperature (20‒22°C), 12-hour light/dark cycle, fed with balanced feed and *ad* libitum water, evaluated for general status and motor conditions, were distributed, by simple draw, into four groups of ten animals each, all submitted to laminectomy and three groups submitted to subsequent spinal cord injury, as described below:

a) Group 1 – Laminectomy + Spinal Cord Injury without intervention – rats submitted to laminectomy and spinal cord injury without any intervention for control;
b) Group 2 – Laminectomy + Contused Spinal Cord Injury + intraperitoneal administration of 0.9% Saline Solution (SS) – rats submitted to spinal cord injury and 0.9% Saline Solution (SS) for comparison with the intervention group – control group/placebo;
c) Group 3 – Laminectomy + Contused Spinal Cord Injury + intraperitoneal administration of GSH (8 mg/kg) – rats submitted to spinal cord injury and intraperitoneal administration of GSH;
d) Group 4 – Laminectomy only, without suffering from spinal cord injury and without any intervention.

The rats were anesthetized with 10 mg/kg of xylazine and 50 mg/kg of ketamine intraperitoneally. For local anesthesia, lidocaine hydrochloride with epinephrine (adrenaline) was used and submitted to antibiotic prophylaxis during surgery with 5 mg/kg of cefazolin sodium (antibiotic) intraperitoneally, immediately before the injury and once a day for the following three days. All rats underwent laminectomy, including those without spinal cord injury; a surgical microscope was used for spinal cord exposure. Then, the rat was positioned in the computerized equipment New York University (NYU) impactor to produce the controlled spinal cord injury, following the established Multicenter Animal Spinal Cord Injury Study (MASCIS) protocol, standardized for Wistar rats.^9^^,^^10^ Groups 1 and 4 received no pharmacological intervention. GSH was administered to the rats in Group 3 by intraperitoneal injection at a dosage of 8 mg/kg immediately after spinal cord injury while the animals were still under anesthesia and sedation. The rats in Group 2 suffered the injury and received an intraperitoneal injection of 0.9% SS. The evaluation of locomotor function was performed following the motor evaluation protocol by the BBB scale on days 2, 7, 14, 21, 28, 35, and 42 after the spinal cord injury and the horizontal ladder test on day 2 postoperative and at 2, 4, and 6 weeks. The rats were euthanized on day 42 after the injury, and then the spine was carefully removed and cut from an extensive dorsal incision from T8 to T12 (about 2.5 cm long). All bone and soft tissue structures adjacent to the spinal cord were removed with a pierced bar until completely exposed. The marrow was identified as follows:

a) Area “A”: Cranial region (proximal) to the injury;
b) Area “B”: Central region with spinal cord injury;
c) Area “C”: Caudal region (distal) to the injury.

The segments of the spinal cord (“A”, “B”, and “C”) underwent two types of histological analysis: tissue injury and axonal regeneration.^11^ For the qualitative analysis, the B-area slides were evaluated for necrosis, hemorrhage, hyperemia, degeneration of the nervous substance, and cellular infiltrate. A score ranging from 0 to 3 (absent, discrete, moderate, and intense) was attributed to the findings in each section of the bone marrow histologically studied.^12^

For the quantitative analysis of the proximal (“A”) and distal (“C”) areas, two fields of each segment were selected and randomly chosen by the pathologist. The photos were analyzed using Sigma Scan Pro 5.0 software for counting axon fibers. Only neurons with a diameter ≥ 15 μm were considered for counting.^13^ The number of regenerated axons in the distal segment and the number of proximal axons were applied to the following formula^12^, ^13^, ^14^ to calculate an axonal Regeneration Index (RI):

RI=(numberofaxonsinthedistalsegment/numberofaxonsintheproximalsegment)×100.

For statistical analysis, the nonparametric Kruskal-Wallis test was used to compare more than two groups, the Mann-Whitney test was used in the paired comparison between two groups, and one-way analysis of variance (ANOVA) was used for the analysis of BBB, cell count, and histological analysis. To analyze the follow-up of each group, the ANOVA test for repeated measures was used. The type I error with p ≤ 0.05 was accepted.

## Results

In the BBB evaluation, from day 7 after spinal cord injury, a statistically significant difference was observed in locomotor function in all four groups (p < 0.001) and the comparison of improvement of motor function (p < 0.001). In addition, statistically significant differences were observed from day 14 (p ≤ 0.05) among the groups. In the weekly evolution among the groups, no statistical difference was found in the first week among Group 1 (laminectomy with spinal cord injury), Group 2 (laminectomy with spinal cord injury and SS), and Group 3 (laminectomy with spinal cord injury and GSH). However, there was a statistical difference between the SS and GSH groups in the second week, which was not maintained in the third and fourth weeks. From the fifth week, there was a statistically significant difference between the GSH and SS groups, with better results in the GSH group.

The horizontal ladder evaluation showed a statistical difference between the SS and GSH groups from the second week, which did not remain in the third and fourth weeks. From the fifth week, there was a statistically significant difference between the GSH and SS groups, with better results in the GSH group.

Tissue evaluation of the spinal cord injury area (B-area) showed degeneration, hemorrhage, hyperemia, cellular infiltrate, and necrosis. The variables were described as absent, discrete, moderate, and intense. Group 4 without spinal cord injury did not present areas of degeneration, hemorrhage, hyperemia, cellular infiltrate, or necrosis, as expected. No statistically significant difference among the groups submitted to spinal cord injury (p > 0.05).

The mean axonal RI demonstrated a statistical difference among the groups added together. Group 3 presented a higher mean RI than groups 1 and 2 and lower than Group 4. The group submitted to intervention with GSH showed statistically significant improvement concerning the other groups submitted to spinal cord injury favorable to intervention with GSH (p < 0.05), and, as expected, statistically significant difference to the group submitted to laminectomy without spinal cord injury in favor of the latter.

## Discussion

There are no medications approved that are capable of reversing neural tissue damage.^15^ Research has sought different strategies to improve functional recovery after spinal cord trauma, focusing on reducing secondary damage and stimulating tissue regeneration. Physical means are used, such as hypothermia, oxygen therapy and exercises, substances that promote the improvement of the inflammatory environment and control of free radicals, and therapies with different cell types to promote neuronal recovery, using other animal models.^16^, ^17^, ^18^, ^19^, ^20^, ^21^, ^22^, ^23^, ^24^, ^25^

Our study used the standardized model to investigate the effects of GSH, an antioxidant, in the experimental spinal cord injury.^26^, ^27^, ^28^, ^29^, ^30^ GSH plays an important role in controlling reactive oxygen species that arise after spinal cord injury and are responsible for the propagation of tissue injury. Different studies demonstrate the GSH in its reduced form when there is injury by oxygen free radicals in different cell types, being even more important in neuronal cells. This substance is inserted in the secondary injury cycle to stop the propagation of cell damage, thus having a neuroprotective effect. Some studies demonstrate this effect in several cell types.^28^, ^29^, ^30^

The difference in the results of the several functional scores evaluated, presenting a statistically significant improvement in the BBB scale and improvement without a statistically significant difference in the horizontal ladder test, may be due to the difference in the scores, with the BBB scale being more detailed to its evaluation. However, both tests are validated in the literature for motor evaluation in experimental studies with rats.

The histological analysis regarding the degeneration, hemorrhage, hyperemia, cellular infiltrate, and necrosis was performed quantitatively to enable statistical analysis. However, this generates some degree of subjectivity for the scores evaluated for each variable. In this analysis, there was no statistically significant difference. On the other hand, the evaluation of neuronal count through the axonal RI was higher in the group submitted to intervention with GSH compared to the other groups submitted to spinal cord injury.

The secondary cellular events that follow the initial traumatic mechanical injury contribute to the propagation of tissue damage associated with cell damage caused by the release of oxygen free radicals and the reduction of mechanisms and substances responsible for maintaining cellular homeostasis, highlighting the role of GSH in its reduced form.^29^^,^^30^ Furthermore, associated with oxidative stress and inflammatory reaction, the harmful effect of vascular and ischemic disorders and homeostasis in this propagation of secondary damage is added, which can also be addressed with different therapeutic strategies, making the approach to spinal cord trauma increasingly multifactorial.^31^, ^32^, ^33^

## Conclusion

This study demonstrated that using glutathione in the experimental spinal cord trauma scenario leads to better statistically significant functional recovery through the BBB score and improvement of the axonal regeneration index in Wistar rats submitted to experimental spinal cord injury.

## Data availability

The authors confirm that the data supporting the findings of this study are available within the article. Furthermore, the data sets used and/or analyzed during the current study are available from the corresponding author upon reasonable request.

## Ethics approval

This study was approved by the Ethics internal review boards of the Biosciences Institute under the number 956/2018.

## Authors’ contributions

Fernando Flores de Araujo: Data curation, forma analysis; Writing - original draft.

Raphael Martus Marcon: Methodology; Project administration; Writing - review and editing.

Alexandre Fogaça Cristante: Resources; Supervision.

Tarcísio Eloy Pessoa Barros Filho: Funding Acquisition; Validation.

## Funding

No funding was received for this project.

## Declaration of competing interest

The authors declare no conflicts of interest.
